# Functional genomics of the pregnant uterus: from expectations to reality, a compilation of studies in the myometrium

**DOI:** 10.1186/1471-2393-7-S1-S4

**Published:** 2007-06-01

**Authors:** Michèle Breuiller-Fouche, Gilles Charpigny, Guy Germain

**Affiliations:** 1 INSERM, U 767, Paris, F-75014, France; 2 INRA, UMR 1198; ENVA; CNRS, FRE 2857, Biologie du Développement et Reproduction, Jouy en Josas, F-78350, France

## Abstract

**Background:**

Studies on the human myometrium have reported on different microarrays containing different sets of genes or ESTs. However each study profiled only a small number of patients due to various constraints. More profiling information would be an addition to our knowledge base of parturition.

**Methods:**

We compiled from five human studies, transcriptional differences between the non pregnant myometrium (NP), preterm myometrium (PTNIL), term myometrium not in labor (TNIL) and term myometrium in labor (TIL). Software modules developed by the Draghici's group at Wayne State University (Detroit, MI, USA) were used to propose a hierarchical list of several KEGG pathways most likely adjusted to changes observed in microarray experiments.

**Results:**

The differential expression of 118 genes could be dispatched in 14 main KEGG pathways that were the most representative of the changes seen in NP and PTNIL, versus TNIL or TIL. Despite the potential of multiple pitfalls inherent to the use of the microarray technology, gene module analysis of the myometrial transcriptome reveals the activation of precise signaling pathways, some of which may have been under evaluated.

**Conclusion:**

The remodelling and maturation processes that the uterus undergoes in pregnancy appear clearly as phenomena which last during the full course of gestation. It is attested by the nature of the main signaling pathways represented, in the comparison of the PTNIL versus TNIL uterus. Comparatively, the onset of labor is a phenomenon which remains less well characterized by these methods of analysis, possibly because it is a phenomenon occurring in too short a window to have been grasped by the studies carried out up to now.

## Background

The common hallmark of recently published papers referring to functional genomics is their enthusiastic tone on expectations. The near-entire genomic sequence of the human and several model animals has given the opportunity to flesh out the relationships between genes, phenotypes and global transcriptional status at unprecedented speed and scale [1]. The microarray technology allows the examination of the function of thousands of genes at once and in parallel, thereby providing an "assay" of the transcriptional status of cells or tissues in a wide variety of physiological or pathophysiological situations [2]. Computational methods allow the identification of genes differentially expressed across cell types, developmental stages or pathological conditions; genes expressed in a coordinated manner across a set of conditions and to delineate clusters of genes sharing coherent expression features [3]. These techniques open up new areas of unrecognized molecular networks and yet unidentified players in the anabolic-catabolic balance in intracellular signaling cascades which have been so far hardly investigated in the context of preterm labor.

From past studies in domestic animals, it has amply been demonstrated that during pregnancy the production/withdrawal of sex steroids, notably that of progesterone which is thought to actively block the transformation of the myometrium to a contractile phenotype, govern the precise timing of labor onset [4]. Steroids modulate transcription by interacting with specific response elements in the promoter regions of target genes to recruit the basic transcriptional complex. Although it is not yet definitively established whether functional progesterone withdrawal is indeed a pivotal event in the human parturition cascade [5], all the preceding elements point to the fact that transcriptome studies of the myometrium, fetal membranes or placenta, should be a valuable approach to decipher the mechanisms involved in human parturition. In the course of the last 6 years, a number of transcriptomic studies on the preterm *versus *term pregnant human uterus [6-12] or fetal membranes [13-17] have been published.

Studies on the human myometrium have reported on different microarrays containing different sets of genes or ESTs. However each study profiled only a small number of patients due to various constraints. More gene expression profiling information would be an addition to our knowledge base of parturition. We compiled from human studies [7-9,12,18], transcriptional differences between the preterm myometrium (PTNIL), term myometrium not in labor (TNIL) and term myometrium in labor (TIL).

## Methods

To translate lists of hundreds of genes differentially regulated in the conditions under study into a clearer understanding, we used combinations of searches through the literature referenced in public databases (Table 1) and the Onto-Tools software developed by the Draghici's group at Wayne State University (Detroit, MI, USA) [19]. The Onto-Express module helps to recognize functional profiles (using gene ontology terms) for the categories: biochemical function; biological process; cellular role; cellular component; molecular function, and chromosome location [20]. The Pathway-Express module helps data mining – proposing on the basis of a computational method a hierarchical list of several KEGG pathways [21,22] most likely adjusted to changes observed in microarray experiments [23,24]. KEGG is a knowledge base for systematic analysis of gene functions, linking genomic information with higher order functional information [25]. Input data files for use with the Onto-Tools software modules were built on gene lists reported in published papers or, when available, on the complete gene expression data set, deposited as supplemental data at a public Internet site [7-9,12,18].

## Results

**Figure 1:**
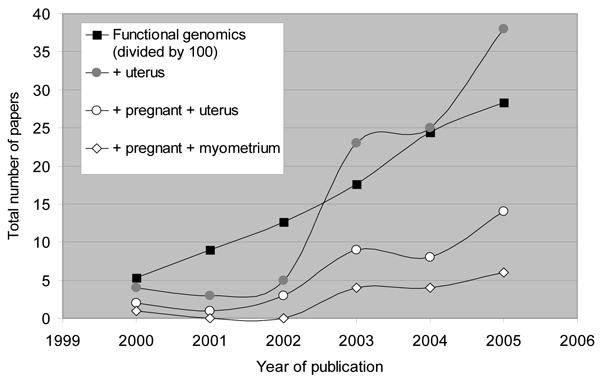
**Number of articles referring to functional genomics in the PubMed database, years 2000 to 2005 [35]**. Keywords for search strategy (limits: human): functional genomics OR microarray, (black squares); (functional genomics OR microarray) AND uterus (closed grey circles); (functional genomics OR microarray) AND uterus AND pregnant (open white circles); (functional genomics OR microarray) AND uterus AND myometrium (open white diamonds). On the graph, the number of papers is divided by 100 for the total papers on functional genomics OR microarray (black squares).

Genomic studies of the uterus remain scarce and accounted for approximately 1% of the total of genomic studies published in the past 6 years. Studies in pregnancy represented about 1/3 of the total studies on the uterus and studies specifically dedicated to the myometrium were even fewer (Figure 1).

In the myometrium, the differential expression of 118 genes could be distributed in 14 main KEGG pathways that were the most representative of the changes seen in NP versus TNIL, or PTNIL versus TNIL or TIL. It is to notice that expression of genes involved in growth and cytoskeletal remodeling of myometrial cells was critically modified in the comparison of the non pregnant versus pregnant uterus, until term. Conversely, limited changes in gene transcription, characterized the transition between the term and term in labor stages in the myometrium. Interestingly, genes involved in apoptosis were only expressed in advanced gestation and their changes clearly anticipated the onset of labor (Table 2).

**Figure 2:**
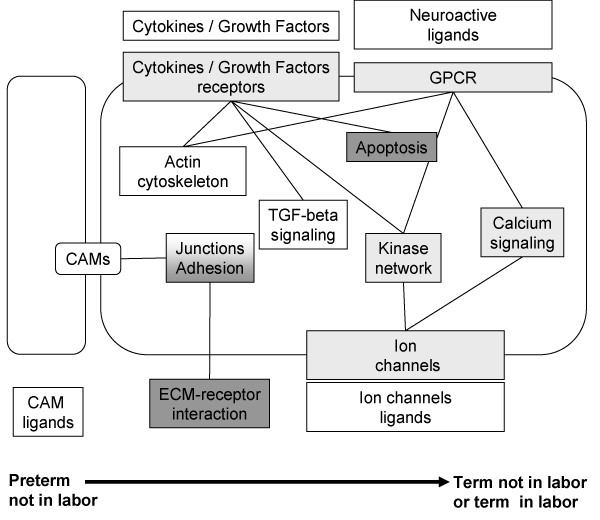
**Main pathways represented in the myometrium on the basis of the genes detected in genomic studies**. Gene transcripts pertaining to these pathways were either up-regulated (dark grey) or down-regulated (light grey) by a two fold or more factor. White boxes denote insufficiently documented links in context. Comparison between Preterm not in labor and Term not in labor or Term in labor. Pathways are shown with their known links to cytokine, growth factors (GF) and G-protein coupled membrane receptors (GPCR). CAMs, cell adhesion molecules. ECM, extra-cellular matrix).

Table 3 lists the genes expressed in the uterus for the MAPK signaling pathway which is a good example of a pathway whose changes remain active until the end of pregnancy. An overall picture of the general trend of changes in gene expression pertaining to the 14 KEGG pathways that characterize the transition of the preterm versus term uterus is summarized in Figure 2.

## Discussion

Drawbacks and limitations inherent to the use of Onto-Tools or of their cognate alternatives for ontological analysis have been discussed. These limitations remain to the present day, questions over the robustness of array data and the criteria under which their conclusions were drawn have been made [23]. A recent review by Allison *et al*. [26], has the merit of proposing simple sound recommendations for future microarrays analysis methods.

Despite the potential of pitfalls inherent to the use of the microarray technology (review in [27]), gene module analysis of the myometrial transcriptome reveals the activation of precise signaling pathways, some of which may have been under evaluated. Thus, the remodelling and maturation processes that the uterus undergoes in pregnancy appear clearly as phenomena which last during the full course of gestation. It is attested by the nature of the main signaling pathways represented, in the comparison of the PTNIL versus TNIL uterus. Comparatively, the onset of labor is a phenomenon which remains less well described by these methods of analysis, possibly because it is a phenomenon occurring in too short a window to have been grasped by the few studies carried out up to now.

Also, although the myometrium is considered to be a relatively homogeneous tissue, some of its largest changes at term occurred in genes that were not normally associated with muscle. This is consistent with animal studies which better allow to explore the global pattern of gene expression and their co-regulation on the basis of their genomic location over the full time-course of myometrial transformation or following experimentally-controlled infection [28-31].

Today, a main handicap with high throughput methods is the capacity for each individual research group to handle samples and data. These methods indeed suggested a number of potentially new biochemical markers of preterm labor [32], some of which, for example, could be detected in circulating immune-cells. However, all studies, notably in the human, have suffered from several limitations, among which are the limited size of samples which hardly can be considered as representative of even a small population; the regular inconsistency in nomenclature usage or terminology in reference to the molecules studied; the lack of integrated databases to compile different studies and the underestimation of heterogeneity of the tissue exposed to the microarray analysis.

## Conclusion

Genomic studies of the pregnant uterus, like in other domains, widen our comprehension of the structural and metabolic transformations which affect the myometrium from the beginning of pregnancy until parturition. Although the studies so far published remain a few numbers, they throw light on the urgent need to constitute a Working Group to standardize the techniques, terminology and experimental plans as well as to design new integrated databasing protocols. In this respect we have much to learn from those who anticipated the burst of genome biology [33]. Access to dedicated databases by researchers and clinicians will be critical to progress in biomarker identification for the major obstetrical concern, namely the preterm birth.

## List of abbreviations

CAMs, cell adhesion molecules; ECM, extra-cellular matrix; ESTs, expressed sequence tags; GF, growth factors; GPCR, G-protein coupled membrane receptors; JAK/STAT, Janus kinases/Signal transducers and activators of transcription; KEGG, Kyoto Encyclopedia of Genes and Genomes; MAPK, mitogen-activated protein kinase; NP, non pregnant; OMIM, Online Mendelian Inheritance in Man; PTNIL, preterm not in labor; TGF, transforming growth factor; TIL, term myometrium in labor; TNIL, term not in labor; Wnt protein/signaling, wingless-type protein/signaling. Gene names, see [34] for detailed names of genes listed in this article.

## Competing interests

The authors declare that they have no competing interests.

## Authors' contributions

MBF carried out the searches through the literature referenced in public databases and helped to draft the manuscript. GC participated in the design of the study and helped to perform the statistical analysis. GG conceived the study, contributed to its statistical analysis and finalized the draft of the manuscript. All authors read and approved the final manuscript.

## Figures and Tables

**Table 1:** Studies used to compile the differential expression of the genes in the myometrium

Author reference	Platform	Number of genes on chip	Supplemental data, where?	Comparison
Rehman *et al*., [9]	Incyte Genomics UniGEM V microarray Version 1.0	7,075 cDNA	- Yes (raw data) - # GSM 6791 NCBI GEO (from 2005)	NP-TNIL

Bethin *et al*., [8]	Affymetrix HG-U95A chips	U95 set, approx. 10,000 genes (oligo)	- Yes - PDF files - no open access	PTNIL-TNIL
Charpigny *et al*., [7]	Clontech Atlas Human 1.2 & 1.2II	2,352 cDNA	- Yes (raw data) - XLS files - Lab., open access	PTNIL-TNIL

Esplin *et al*., [18]	Unspecified	6,912 cDNA in duplicate	- Yes (raw data) - XLS files - Lab., open access	TNIL-TIL
Havelock *et al*., [12]	Incyte Genomics UniGEM V microarray Version 2.0	9,182 cDNA	- No	TNIL-TIL

NP-TNIL, Non-pregnant (NP) versus term not in labor myometrium (TNIL); PTNIL-TNIL, preterm not in labor (PTNIL) versus TNIL myometrium; TNIL-TIL, TNIL versus term in labor myometrium (TIL).

**Table 2:** The main KEGG metabolic pathways, regulatory pathways or molecular complexes expressed in the myometrium

KEGG pathways^1^	NP-TNIL	PTNIL-TNIL	TNIL-TIL
Actin cytoskeleton	15	6	0
Focal adhesion	18	6	3
Adherens junctions	11	3	0
Tight junctions	10	0	0
Cytokines-cytokine receptors	8	12	5
Toll-like receptors	5	6	1
TGF-beta signaling	6	2	3
Wnt signaling	7	2	1
MAPK signaling	15	12	4
Phosphatidylinositol signaling	5	8	0
JAK/STAT signaling	6	7	1
Apoptosis	0	6	0
Calcium signaling	8	8	0
Neuroactive ligand-receptors	6	6	0

^1^A detailed description of KEGG pathways is accessible at [25]. For each pathway, gene transcripts showed at least a 2 fold or more, up or down-regulation after paired comparisons of the following stages: non-pregnant (NP) versus term not in labor myometrium (TNIL), preterm (PTNIL) versus TNIL myometrium, or TNIL versus term in labor myometrium (TIL). Number of genes represented in a defined pathway. A total of 118 differentially expressed genes (genes may belong to several pathways) were detected, 0: no gene represented for the corresponding pathway.

**Table 3:** The MAPK signaling pathway differentially expressed in the myometrium

MAPK signaling pathway		
NP-TNIL^1 ^(15)	PTNIL-TNIL^2 ^(12)	TNIL-TIL^3 ^(4)

CD14(+)	EGF(+)	BDNF(+)
EGFR(+)	IKBKB(+)	DUSP5(+)
FGFR1(+)	MAP2K4(+)	NR4A1(+)
FLNA(+)	MAPK10(+)	TGFB2(+)
HSPA5(+)	PLA2G5(+)	
HSPA8(+)	RAP1A(+)	
MAP3K5(+)	RASA1(+)	
NLK(+)	TNF(+)	
PLA2G2A(+)	TP53(+)	
PRKCB1(+)	PLA2G2A(-)	
PRKCG(+)	PPP5C(-)	
RAC2(+)	TNFRSF6(-)	
RASGRP2(+)		
RRAS(+)		
YWHAZ(+)		

Genes detected in genomic studies according to ^1^NP-TNIL, to ^2^PTNIL-TNIL or to ^3^TNIL-TIL comparisons, during the cycle or pregnancy. Brackets: number of genes represented in the pathway. All gene transcripts showed at least a 2-fold or more, up (+) or down-regulation (-) after paired comparisons of the following stages: non-pregnant (NP) versus term not in labor (TNIL), preterm (PTNIL) versus TNIL, or TNIL versus term in labor (TIL). OMIM nomenclature [34] adopted for gene names.
